# Prevalence of menopausal symptoms and attitudes towards menopausal hormone therapy in women aged 40–60 years: a cross-sectional study

**DOI:** 10.1186/s12905-023-02621-8

**Published:** 2023-09-04

**Authors:** Jie Lu, Kangfen Li, Xinlie Zheng, Ran Liu, Min Chen, Jingyun Xian, Suhua Tu, Lingling Xie

**Affiliations:** 1 School of Nursing, Southwest Medical University, Sichuan, China; 2 Department of Gynecology, Affiliated Hospital of Southwest Medical University, Sichuan, China; 3 Nursing Department, Affiliated Hospital of Southwest Medical University, Sichuan, China

**Keywords:** Menopause, Menopausal hormone therapy, Physical examination

## Abstract

**Background:**

Menopause is a specific physical and psychological transition period for women, during which they experience a series of menopausal symptoms. Menopausal hormone therapy is an important treatment for improving menopausal symptoms. Helping women correctly understand menopausal hormone therapy is a prerequisite for increasing the acceptance and utilization of menopausal hormone therapy by women. Physical examinations are an important method for women to master their own health status and detect potential health problems, and in recent years, an increasing number of women have actively participated in physical examinations. Therefore, this study aims to comprehend the prevalence of menopausal symptoms and attitudes towards menopausal hormone therapy among women aged 40–60 who underwent physical examinations, which would provide a useful reference to reduce the prevalence of menopausal symptoms and improve acceptance of menopausal hormone therapy.

**Methods:**

This cross-sectional study was conducted at the Health Management Centre of the Affiliated Hospital of Southwest Medical University in Luzhou City, Sichuan Province. The data were collected from 295 women aged between 40 and 60 using convenience sampling. Information on all participants was collected through face-to-face interviews. Participants completed a demographic questionnaire and an attitude towards menopausal hormone therapy questionnaire, and the modified Kupperman index was used to assess the prevalence and severity of participants' menopausal symptoms. The collected data were processed using SPSS and Excel software and analysed using descriptive statistics and logistic regression.

**Results:**

The top 5 menopausal symptoms were insomnia, fatigue, bone and joint pain, sexual dysfunction and emotional instability. Multiple linear regression analysis showed that residence, sexual intercourse frequency, mentality, and physical exercise were the influencing factors of menopausal symptoms. The study showed that 77% of women said they were still reluctant to receive menopausal hormone therapy after experiencing menopause-related symptoms. The main source of menopausal hormone therapy-related knowledge among women was from surrounding menopausal women (62%), and 54% wanted to gain menopausal hormone therapy-related knowledge through a web-based approach.

**Conclusion:**

The incidence of menopausal symptoms is higher in women aged 40–60 years, which is related to women’s mentality, exercise, and sexual intercourse frequency. In addition, the results of this study indicate that women’s knowledge of menopausal hormone therapy is insufficient, which suggests that we need to strengthen health education to improve the acceptance rate of menopausal hormone therapy.

## Introduction

Menopause is the reproductive to nonreproductive transition period in women and is a necessary period in the life of a woman [1]. Because of the decline in ovarian function, hormone levels can fluctuate dramatically, which can cause women to experience hot flashes, insomnia, depression, and other related symptoms [2–4], and these physical and mental symptoms can lead to a reduction in a woman’s health level and quality of life [5, 6].In addition, postmenopausal women have an increased risk of osteoporosis [7] and genitourinary syndrome [8].

Menopausal hormone therapy (MHT) is a common treatment to improve menopausal symptoms and can effectively reduce the adverse effects caused by hormone level fluctuations [9]. A randomized controlled trial that included 150 women showed that MHT improved hot flashes and enhanced women’s quality of life [10]. In addition, several studies have confirmed the benefits of MHT for menopausal women; for example, MHT can improve hot flashes in menopausal women [11] and reduce the incidence of osteoporosis [12] and cardiovascular disease [13]. In 2016, the International Menopause Society suggested that MHT is an important step in improving the health of menopausal women [14]. This shows that the benefits of MHT as an important treatment are widely recognized. Women in China have fewer opportunities to receive menopausal health education, have a single form of health education, and know less about menopause and MHT, so they do not choose to receive MHT after menopausal symptoms appear and let nature take its course. The results of a research study showed that fewer menopausal women in China actively seek medical treatment and have a lower willingness to undergo MHT [15]. It is clearly stated in the Endocrine Society Clinical Practice Guidelines [16] that MHT needs to be individualized according to a standardized diagnosis and treatment process, and women receiving MHT should receive corresponding health guidance to develop better management strategies. Therefore, how to increase the acceptance rate of MHT among women is an urgent issue that needs to be addressed.

In recent years, with the economic development of China and the support of national policies, women’s awareness of self-care has been increasing, and an increasing number of women are actively participating in physical examinations. Currently, research on menopause has been conducted in various populations. For example, in community groups of women, the prevalence of menopausal symptoms is as high as 73.8% [15]. Additionally, a study focused on rural women in India indicated that perimenopausal women are more prone to experiencing menopausal symptoms compared to pre-menopausal and post-menopausal women [17]. However, there is a dearth of menopausal studies on women undergoing physical examinations.

Therefore, through this cross-sectional study, we aim to comprehend the prevalence of menopausal symptoms among women undergoing physical examinations, along with their attitudes towards menopausal hormone therapy, so as to inform the reduction of the prevalence of menopausal symptoms and improve acceptance of menopausal hormone therapy.

## Methods

### Study design

This is a cross-sectional study. We selected respondents using the convenience sampling method and chose women who had undergone physical examinations at the Health Management Centre of the Affiliated Hospital of Southwest Medical University from March 2022 to August 2022.

### Inclusion and exclusion criteria

The inclusion criteria were as follows: 1. Women aged 40–60 years old; 2. Women who were able to understand and cooperate with the study; and 3. Women who provided informed consent. The exclusion criteria were as follows: 1. Patients with heart, liver, lung, kidney, brain and other major organ diseases; 2. Patients with malignant tumours; and 3. Patients with mental health conditions that could affect their cooperation. The study was approved by the Clinical Trial Ethics Committee of the Affiliated Hospital of Southwest Medical University (Approval No.: KY2022083), and the study subjects gave informed consent and volunteered to participate in this study.

### Sample size determination and calculation

The sample size for this study is based on the single population proportion formula. The specific formula is as follows: $\mathrm n=\;\frac{\left({\mathrm Z}_{\mathrm a/2}\right){}^2\mathrm P\left(1-\mathrm P\right)}{\mathrm d^2}$. In this sample size formula, we set the confidence interval to 95%, the value of zα/2 to 1.96, the expected prevalence of menopausal symptoms among Chinese women aged 40–60 years to 80% [18], the *P* value to 0.8, and the marginal sampling error tolerated to 0.05, and substituting into the formula, we obtained: $\mathrm n\;=\;\frac{\left(1.96\right)^2\;0.8\;\left(1-0.8\right)}{\left(0.05\right)^2}$. In addition, considering the 10% non-response rate, we finally confirmed the sample size of 271.

### Sampling procedures

**Fig. 1:**
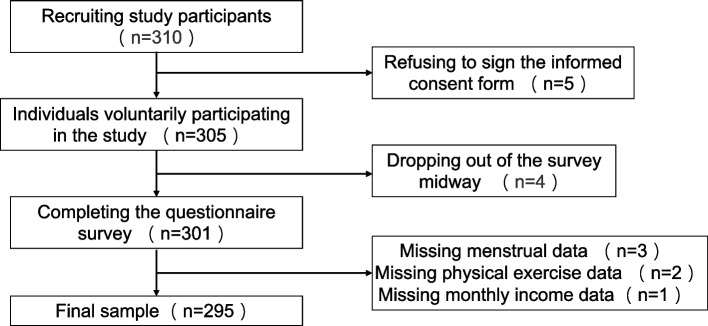
Flow chart of participant selection

The flow chart of participant selection is shown in Fig. 1. A total of 310 participants who met the inclusion and exclusion criteria were recruited for this study, of whom five refused to sign the informed consent form, four dropped out of the survey midway, and six missed important data. Finally, the number of valid questionnaires in this study was 295.

### Measurement

We administered face-to-face interviews to all participants. The investigation content was as follows: 1. A demographic questionnaire: This questionnaire collected data on age, residence, education background, marital status, career, monthly income, menstrual condition, taking hormonal medication, mentality, sexual intercourse frequency, number of children and physical exercise level;2. The modified Kupperman index: The modified Kupperman Scale index was used to assess the severity of menopausal symptoms, which has been shown to be suitable for Chinese women [19]. The scale included the assessment of 13 common menopausal symptoms: hot flashes and sweating, insomnia, mood swings, urinary tract infections, sensory disturbances, skin tingling, depression, headache, palpitations, dizziness, sex life status, bone and joint pain, and fatigue. The basic score for each symptom varied, with hot flashes and sweating being 4 points, insomnia, mood swings, and sensory disturbances being 2 points, and the remaining symptoms being 1 point. According to the severity of symptoms, there were four-degree scores: normal (0 points), mild (1 point), moderate (2 points), and severe (3 points). The total score for each symptom was calculated by multiplying the basic score by the degree score, and the sum of all the symptom scores was the total score of the scale, with scores ranging from 0 to 63 points; scores ≤ 6 points indicated normal symptoms, 7 to 15 points indicated mild symptoms, scores of 16 to 30 points indicated moderate symptoms, and scores > 30 points indicated severe symptoms; 3. An attitude towards menopausal hormone therapy questionnaire: This questionnaire includes understanding of menopause, understanding of MHT, sources of knowledge related to MHT, desired health education methods, willingness to use MHT after symptoms, reason for receiving MHT, and reason for refusing MHT.

### Data quality control

First, in the research design stage, we chose a validated scale that is more applicable to Chinese women as the survey instrument [19]. Second, before the survey began, we provided uniform and standardised training to the investigators to ensure the reliability and validity of the questionnaire. Finally, before data entry, the researchers scrutinised the collected data. During the data entry process, it was carried out independently by two researchers, and data verification was performed using Excel software to ensure the accuracy of the data.

### Statistical analysis

We used Excel software for data entry and charting, and we performed descriptive and logistic regression analyses with the help of SPSS software. In analysing the descriptive statistics, we used the mean and standard deviation, and the distribution was expressed using frequency and percentage. While exploring the influencing factors, we performed a logistic regression analysis considering several variables such as residence, mentality, number of children, sexual intercourse frequency, taking hormonal medication, and physical exercise. The test level was set at α = 0.05.

## Results

### General demographic characteristics

**Fig. 2:**
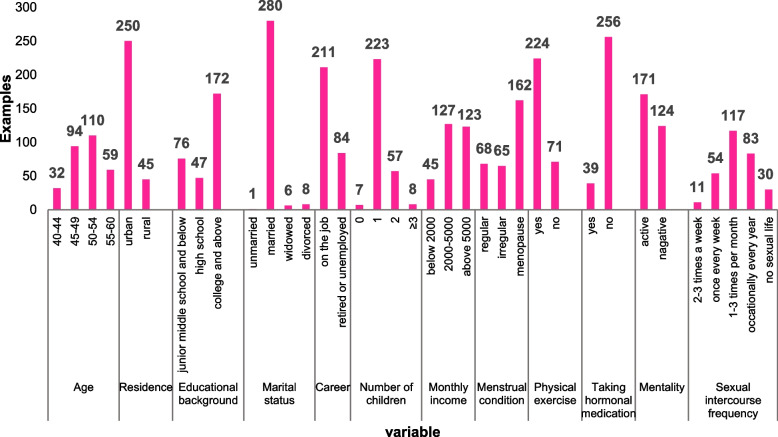
General demographic characteristics

The age range of the respondents was predominantly from 45–54 years old (69.15%), most women had a college level education or above (58.31%), were employed (71.50%), and had a monthly income ranging from ¥2000 to 5000 (43.10%) Yuan. The investigated women had an average menarche age of 13.25 years (SD ± 1.24) and an average menopause age of 48.98 years (SD ± 3.29) (Fig. 2).

### Occurrence of menopausal symptoms

**Fig. 3:**
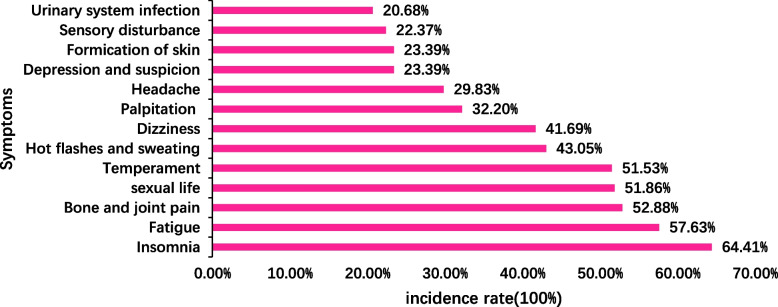
Incidence of menopausal symptoms (*n* = 295)

**Fig. 4:**
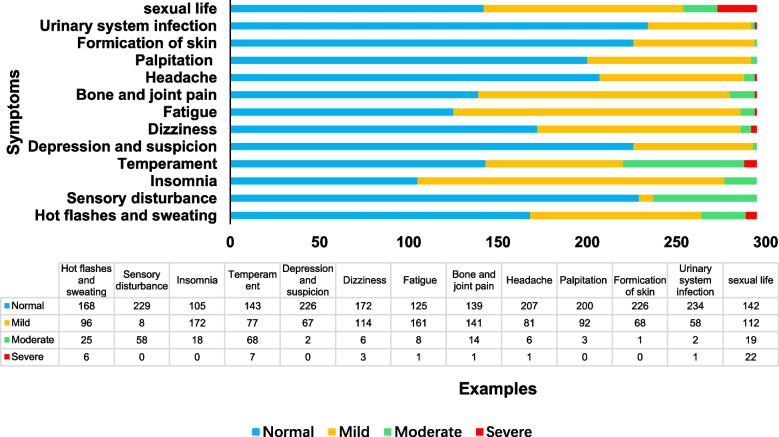
Distribution of menopausal symptoms (*n* = 295)

Eighty-seven respondents had no symptoms, and 208 had menopausal symptoms. The top five menopausal symptoms were insomnia, fatigue, bone and joint pain, sexual dysfunction and emotional instability. The severity of menopausal symptoms is shown in Table 1, the incidence of menopausal symptoms is shown in Fig. 3, and the distribution of menopausal symptoms is shown in Fig. 4.

**Table 1:** Severity of symptoms during menopause

Degree of symptoms during menopause	Number (*n* = 295)	Percentage	Modified kupperman Score
Normal	87	29.49	3.64 ± 1.91
Mild	137	46.44	10.59 ± 2.43
Moderate and Severe	71	24.07	19.72 ± 3.47

### Multivariate analysis of the modified Kupperman score for menopausal symptoms

**Fig. 5:**
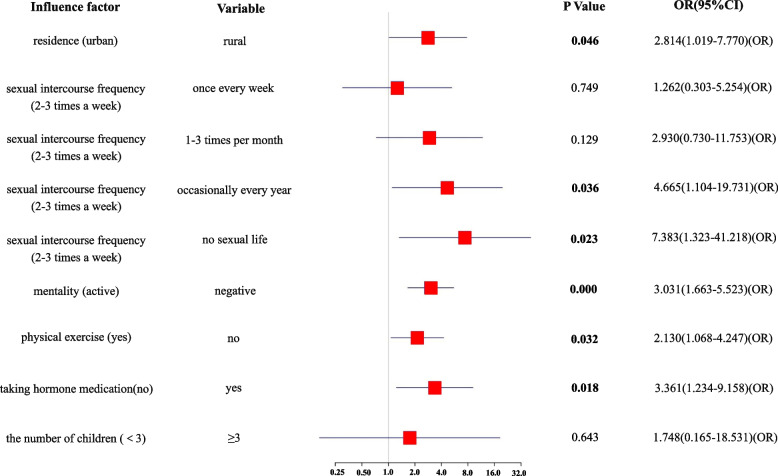
Multivariate logistic regression analysis of menopausal symptoms

Multivariate logistic regression analysis was performed with the modified Kupperman score for menopausal symptoms as the dependent variable and residence, mentality, the number of children, sexual intercourse frequency, the use of hormonal medications, and physical exercise as the independent variables, and the results showed that residence in a rural area, emotional instability, a low sexual intercourse frequency, the use of hormonal medications, and a lack of physical exercise were risk factors for menopausal symptoms (Fig. 5).

### Analysis of cognitive status related to menopausal hormone therapy

The survey results showed that 22% of women had a complete understanding of MHT, MHT-related knowledge was mainly obtained from surrounding menopausal women, and more than half of the women wanted to obtain MHT-related knowledge through an internet-based approach. A total of 23.05% were willing to undergo MHT after experiencing menopausal symptoms, and improving menopausal symptoms was the primary reason why women were willing to accept MHT. The primary reason why women refused MHT was that they considered menopause to be natural (Table 2).

**Table 2:** Cognitive status of menopausal hormone therapy (*n* = 295)

Category	Frequency	Ratio
Understanding of menopause		
Understand	64	0.22
Partial understand	218	0.74
Not understand	13	0.04
Understanding of MHT		
Understand	13	0.04
Partial understand	67	0.23
Not understand	215	0.73
Sources of knowledge related to MHT		
Surrounding Menopausal women	182	0.62
Hospitals, community health service centers, clinic	123	0.42
WeChat public platform, Internet hospital, Search engine, Mobile phone SMS	115	0.39
Television, radio, broadcast	29	0.10
Related books, newspapers, magazines, promotional brochures, Journals	20	0.07
Desired health education methods		
WeChat public platform, Internet hospital, Search engine, Mobile phone SMS	159	0.54
Professional lectures provided by medical units or residential/village committees	85	0.29
Television, radio, broadcast	68	0.23
Related books, newspapers, magazines, promotional brochures, journals	64	0.22
Willingness to use MHT after symptoms		
Willing to	68	0.23
Not willing	227	0.77
Reason for receiving MHT (*n* = 68)		
Improve menopausal symptoms	68	1.00
Prevention of cardiovascular and cerebrovascular diseases	27	0.40
Preventing osteoporosis and fractures	10	0.15
Maintain body shape and skin elasticity	7	0.10
Delaying Alzheimer’s disease and maintaining ovarian function	4	0.06
Reduce the risk of diabetes and Alzheimer’s disease	1	0.01
Reason for refusing MHT (*n* = 227)		
Menopause should be natural	186	0.82
Worried about increasing cancer risk	37	0.16
Worried about increasing the incidence of cardiovascular and cerebrovascular diseases	31	0.14
Worried about increasing the risk of obesity	23	0.10
Worried about causing abnormal vaginal bleeding	16	0.07

## Discussion

Menopausal symptoms are unique abnormal symptoms in women and important factors affecting women’s physical and mental health and living standards. We found that the incidence of menopausal-related symptoms in women who underwent physical examinations was high, but to a lesser extent; this was basically consistent with the results of Ding et al. [20] study carried out in nurses but slightly lower than the results of Huang et al. [18] survey carried out in menopausal clinics in Shanghai, which may be related to different cultural backgrounds, economic levels, and populations selected for the survey. The occurrence of menopausal symptoms differs in women in different regions: surveys in Cambodia have found that the most prevalent menopausal symptom is fatigue [21]. A study carried out in Zhejiang found that bone and joint pain is the most common symptom in menopausal women [22]. Hot flashes, insomnia, bone and joint pain, mood swings, and palpitations are the reasons why Chinese women seek medical treatment [23], while in Western countries, vasomotor symptoms (e.g., hot flashes and sweating) are the most common symptoms in menopausal women and one of the most important reasons women seek medical treatment [24]. In addition, a study in the United States noted that hot flashes lasted for an average of 7.4 years in women [3]. In this survey, the top five menopausal symptoms were insomnia, fatigue, bone and joint pain, sexual dysfunction, and emotional instability. It is suggested that medical staff should pay attention to symptoms with higher incidence and implement targeted coping strategies in combination with the characteristics of each region.

We analysed the influencing factors of menopausal symptoms and found that women living in urban areas, with a positive mentality, a high sexual intercourse frequency, and regular physical exercise had a lower incidence of menopausal-related symptoms, which was consistent with the findings of Wang [25] and AN [26]. Women living in rural areas have fewer opportunities to receive menopause-related health education, have less understanding of the menopausal period, and cannot seek medical attention in a timely manner when menopausal symptoms occur, which in turn increases the possibility of menopausal symptoms [27]. In addition, maintaining a good mentality during the menopausal period is an important factor in reducing the occurrence of menopausal syndrome. The more positive the attitudes of women towards the menopausal period, the more likely they are to actively seek help from others to obtain more mental and material support after the onset of menopausal symptoms, and the more able they are to cope with the physical and mental changes brought about by the menopausal period. Physical exercise is a promoting factor for women to maintain their physical health and plays an important role in preventing and improving menopausal symptoms [28]. Regular physical exercise can improve sleep quality, help to maintain a good mood, and prevent obesity and has a positive effect on reducing the occurrence of menopausal symptoms in women. Studies have shown that there is a positive and significant relationship between menopausal symptoms and social support, and menopausal symptoms decrease with increasing social support [29]. Spousal support is an important source of social support, and sexual intercourse frequency can to a certain extent reflect whether the relationship between husband and wife is harmonious. A good relationship between a husband and wife can improve spousal support for women and can effectively alleviate the adverse psychological emotions caused by the menopausal period, thereby improving women’s well-being and relieving their menopausal symptoms. In summary, maintaining a good positive mentality, engaging in reasonable physical exercise, and promoting a good marital relationship during the menopausal period are beneficial in reducing the incidence of menopausal symptoms. The present study found no significant correlation between age, marital status, career, the number of children, and menstruation regulation and the occurrence of menopausal symptoms through univariate analyses, which differed from the results of studies in other countries. A Turkish study noted that women with a menopausal age of 44–50 years had a higher risk of developing menopausal symptoms [30]; a survey among women in the community showed that marital status was one of the factors affecting menopausal symptoms [31]; the results of a Greek study showed that women who were employed had less severe menopausal symptoms [32]; an Iranian study noted that women with a high number of children had more severe menopausal somatic symptoms [33]; and a Sri Lankan study noted that postmenopausal women had more severe menopausal symptoms than premenopausal women [34]. The differences between the results of the foreign studies and the results of the present survey may be due to differences in national policies and the demographic characteristics of the respondents; for example, the one-child policy was implemented in 1979 in China [35], so 76% of the women in the present survey had only one child; in addition, 95% of the respondents in the present study were married, which lacked comparability.

Menopause is a completely new stage in women’s lives, and women’s understanding of menopause directly affects their attitudes towards menopause. Women who lack knowledge of menopause are more likely to have negative attitudes towards it. Research suggests that the severity of menopausal symptoms affects women’s attitudes towards menopause, with fewer symptoms being associated with more positive attitudes towards menopause [36]. A survey of perimenopausal women conducted in the UK showed that more than 60% of women had no understanding of menopause at all [37], whereas Italian studies indicated that more than half of women had never received menopause-related health education [38]. Unlike the results of international surveys, most women in this study had some knowledge of menopause. This result may be due to the high educational level of the respondents in this study, and the respondents were people who came to the hospital for physical examinations, had a high awareness of self-care and received more menopause health education.

MHT is a common treatment to improve menopausal symptoms and can effectively reduce adverse effects caused by fluctuations in hormone levels [39]. The results of this survey showed that the awareness and acceptance rates of MHT were low among the women, which may be related to a low educational level, a lack of knowledge about MHT and relatively higher treatment costs. Studies have shown that negative attitudes toward MHT among healthcare professionals are a significant contributor to lower rates of MHT utilization [40]. An Israeli study found that the majority of women presenting with menopausal symptoms had not received treatment and only 12.6% had received MHT [41], in general agreement with the findings of this study. The results of a Swedish survey showed that most women refused MHT because they considered menopause to be a normal physiological process, and received MHT because they experienced significant menopausal symptoms [42], which is consistent with the present findings. In addition, the results of this survey showed that the main sources of MHT-related knowledge among women were menopausal women around them, medical units, and internet-based pathways; 42% of the respondents’ knowledge originated from medical units, and most of the respondents’ menopause-related knowledge came from nonmedical units. The results of a cross-sectional survey conducted in Brazil showed that the main sources of women’s knowledge about menopause were close friends, family members, and relatives, which is consistent with the results of this study [43]. In recent years, internet information technology has developed rapidly, and the internet has the advantages of convenience, privacy, promotion, etc., so that women can receive menopause-related knowledge anytime and anywhere [44]. This study showed that more than half of the women wanted to gain knowledge about MHT through a web-based approach. At present, the internet has become one of the important ways for women to obtain MHT related knowledge in China, but the quality of information sourced from the internet varies and some of it has a commercial purpose [44, 45], resulting in women not being able to access scientific information. According to a Canadian study, 17% of website information resources were rated as having very poor information quality, incomplete information and inaccurate information in the search results for “MHT” [46]. In addition, the readability of information on websites is important to the reader, and studies have found that information on websites about menopause is poorly readable and, in parts, difficult to understand [47]. Therefore, it is particularly important to improve the scientific content of internet resources. Relevant departments should strengthen the supervision of internet information and build an internet information platform that is managed by medical and health professionals to ensure the scientific accuracy of information.

In addition, professional guidance by health care professionals is one of the important factors affecting whether women are willing to accept MHT [48], the study found that women who had a discussion with a healthcare professional were more willing to undergo MHT [49]. A study of Jamaican primary care physicians and gynecologists found that 66% were knowledgeable about treatment options for menopause and that the more knowledgeable a physician was, the more likely he or she was to direct patients to MHT treatment [50]. But the current status of healthcare professionals’ attitudes towards MHT and mastery of related knowledge is not good. The results of a questionnaire survey carried out in China showed that among 3216 clinical staff members, 23.3% believed that MHT was not necessary [51]. In a survey of 3426 healthcare professionals, 44.7% had not participated in professional menopausal management training in the past year, and nearly half of them could not correctly identify contraindications to MHT [52]. Therefore, to improve the awareness, acceptance and usage rates of MHT, it is necessary to strengthen the training of medical and health professionals and improve their knowledge levels.

### Strengths and limitations

Our study has several key strengths. First, the data collection method of face-to-face interviews enabled us to collect a reliable dataset that reflected the population of the study. Second, the use of a validated questionnaire ensured the reliability of the data collected. Finally, we converted the survey data into bar charts and forest plots to improve the visibility of the data. Our study has some limitations: First, this is a cross-sectional study that can only obtain data on women’s health over a specific time period and cannot track future changes. Cross-sectional studies also cannot indicate causality. Second, we obtained the study data from a single hospital, and the findings may not be generalizable to all women. Third, this study did not collect data on the hormone levels of the survey respondents and cannot analyse and discuss the hormone levels of menopausal women.

### Practice implications

Although our study has some limitations, it has important practical implications in terms of reducing the incidence of menopausal symptoms and improving acceptance of menopausal hormone therapy. Firstly, this study analysed the factors affecting menopausal symptoms, which can inform healthcare providers to adopt targeted interventions to reduce the occurrence of menopausal symptoms. Secondly, based on the results of the study, healthcare providers can make use of the Internet to develop more targeted health education programmes to help women learn about MHT, thereby changing women’s attitudes towards MHT and increasing the acceptance rate of MHT.

## Conclusions

In summary, the higher incidence of menopausal symptoms in women aged 40–60 is related to women's mentality, exercise, and sexual frequency. Therefore, healthcare professionals should help women maintain a positive mindset and moderate physical activity so as to reduce the incidence of menopausal symptoms. In addition, we found that women's knowledge of MHT was insufficient, which suggests that it is necessary for healthcare professionals to improve the health education of MHT in order to increase the rate of knowledge and acceptance of MHT. In order to reduce the incidence of menopausal symptoms, future studies can explore effective interventions by conducting randomised controlled trials. In addition, in order to gain a more in-depth understanding of women's attitudes towards menopausal MHT, future studies may consider conducting qualitative studies.

## Data Availability

The datasets generated and/or analysed during the current study are available from the corresponding author on reasonable request.

## Abbreviation

MHT: Menopausal hormone therapy
